# Pharmacological properties of *Polygonatum* and its active ingredients for the prevention and treatment of cardiovascular diseases

**DOI:** 10.1186/s13020-023-00871-0

**Published:** 2024-01-02

**Authors:** Hongyuan Lin, Wenhui Wang, Mengqi Peng, Yifan Kong, Xiaowei Zhang, Xiaohong Wei, Hongcai Shang

**Affiliations:** 1 College of Integrated Chinese and Western Medicine, Hunan University of Chinese Medicine, Changsha, 410208 China; 2 Weifang Medical University, Weifang, 261000 China; 3 Key Laboratory of Chinese Internal Medicine of Ministry of Education and Beijing, Dongzhimen Hospital, Beijing University of Chinese Medicine, Beijing, 100700 China

**Keywords:** *Polygonatum*, Cardiovascular diseases, Myocardial fibrosis, Anti-inflammation, Anti-oxidative stress, Atherosclerosis

## Abstract

Despite continued advances in prevention and treatment strategies, cardiovascular diseases (CVDs) remain the leading cause of death worldwide, and more effective therapeutic methods are urgently needed. *Polygonatum* is a traditional Chinese herbal medicine with a variety of pharmacological applications and biological activities, such as antioxidant activity, anti-inflammation, antibacterial effect, immune-enhancing effect, glucose regulation, lipid-lowering and anti-atherosclerotic effects, treatment of diabetes and anticancer effect. There has also been more and more evidence to support the cardioprotective effect of *Polygonatum* in recent years. However, up to now, there has been a lack of comprehensive studies on the active ingredients and their pharmacotoxicological effects related to cardiovascular diseases. Therefore, the main active components of *Polygonatum* (including Polysaccharides, Flavonoids, Saponins) and their biological activities were firstly reviewed in this paper. Furthermore, we summarized the pharmacological effects of *Polygonatum*’s active components in preventing and treating CVDs, and its relevant toxicological investigations. Finally, we emphasize the potential of *Polygonatum* in the prevention and treatment of CVDs.

## Background

Despite considerable progresses in prevention and treatment, cardiovascular diseases (CVDs) remain the leading cause of morbidity and mortality globally, seriously threatening to human health [1, 2]. The incidence of CVDs has been increasing in recent years and is predicted to rise to 23.6 million by 2030 [3–5]. The traditional Chinese medicine (TCM) have been applied in the prevention and treatment of CVDs with a long history, according to the therapeutic methods and concepts of promoting blood circulation, dissipating blood stasis, detoxifying, dredging collaterals and tonifying qi [6, 7]. Thousands of years of clinical practice have confirmed the effectiveness of Chinese herbal medicine in treating CVDs [8].

*Polygonatum*, a traditional Chinese herbal medicine, belongs to the genus *Polygonatum* in the plant family *Liliaceae*. The medicinal portions of *Polygonatum* are the dried rhizomes of *Polygonatum kingianum*, *Polygonatum sibiricum*, and *Polygonatum cyrtonthema*. As a natural product resource of the same origin as medicine and food, *Polygonatum* was originally published in Mingyi Bielu. *Polygonatum* is considered to have the effects of tonifying qi and nourishing yin, fortifying spleen, moistening lung, and benefiting kidney, according to the Chinese Pharmacopoeia [9].

*Polygonatum* contains a variety of active chemical ingredients, such as polysaccharides, steroidal saponins, flavonoids, triterpenoid saponins, alkaloids, lignans, coumarins, fatty acids, and aliphatic long-chain compounds [10, 11]. Among the multiple ingredients, *Polygonatum sibiricum* polysaccharides (PSPs) are abundant and hold significant medicinal value. Extensive research has been conducted on PSPs, which are recognized as a highly significant chemical ingredient of *Polygonatum*. A large amount of evidence has demonstrated that *Polygonatum* and its active ingredients have various pharmacological effects, such as antioxidant properties [12], anti-aging effects [13], immunomodulatory effects [14], antibacterial and anti-inflammatory activities [15, 16]. In addition, studies have also proved its efficacy in the prevention and treatment of cardiovascular diseases (CVDs) [17, 18], Alzheimer's disease [19, 20], diabetes [21], cancer [22], etc.

Numerous studies in vitro and in vivo have shed light on the potential benefits of *Polygonatum* in CVDs [23, 24]. Evidence suggests that *Polygonatum* may exert anti-atherosclerotic effects, protect cardiomyocytes, and attenuate myocardial fibrosis, which are achieved through the inhibition of oxidative stress, modulation of inflammatory processes, and regulation of lipid metabolism pathways [18, 25]. PSPs, the active ingredients in *Polygonatum*, has been reported to inhibit oxidative stress to mitigate D-galactose(D-gal)-induced cardiac damage and aging [18]. *Polygonatum* and its active ingredients also have anti-atherosclerotic effects in Apolipoprotein E (ApoE) gene knocked-out mice by inhibiting Toll-like receptor 4 (TLR4)-mediated activation of nuclear factor kappa-B (NF-κB) [25].

The diverse medicinal uses of *Polygonatum* have attracted significant attention, and researchers have focused their attention on its potential bioactive ingredients and pharmacological mechanisms. However, few researches have addressed the phytochemical composition of *Polygonatum* and its pharmacological properties in CVDs in recent years. This paper reviews the protective effects of *Polygonatum* in CVDs and its active ingredients in order to promote the pharmacological research, development, and utilization of *Polygonatum*.

### Main chemical constituents of *Polygonatum* and their biological activities

*Polygonatum* possesses a diverse array of chemical constituents, and researchers have now isolated and identified its composition as polysaccharides, flavonoids, saponins, alkaloids, lignans, and many other types of chemical constituents from its main medicinal parts. It is actually true that *Polygonatum* has different origins, varieties, and chemical compositions.

### Polysaccharides

Polysaccharides are the predominant chemical constituents in *Polygonatum*, comprising a variety of monosaccharides. In a study conducted by Zhao et al. [14], the polysaccharide compounds extracted from *Polygonatum* were found to be primarily composed of fructose and glucose. After optimizing the hydrolysis and analytical methods for PSPs, Zhao et al. concluded that PSPs are mainly composed of fructose, galactose, and galacturonic acid, as well as small amounts of glucose, arabinose, rhamnose, and xylose [26]. Mannose is also considered to be one of the ingredients of PSPs due to the hydrolysis of fructose into mannose and glucose under strong acidic conditions. Hu et al. used ultra-high-performance liquid chromatography quadrupole trap tandem mass spectrometry to analyze the PSPs after hydrolysis under strongly acidic conditions, which included monosaccharides such as glucose, mannose, rhamnose, galactose, ribose, and arabinose [27]. The identified compounds of PSPs are listed in Table 1.

**Table 1:** The polysaccharides isolated from *Polygonatum*

NO	Chemical ingredients	Chemical formula	Source	Extraction methods	References
1	Fructose	C_6_H_12_O_6_	*Polygonatum*	Soaked in 95% ethanol for 2 days, and then extracted with boiling distilled water for 2 h	Zhao, Zhao, Hu [14, 26, 27]
2	Glucose	C_6_H_12_O_6_	*Polygonatum*	Soaked in 95% ethanol for 2 days, and then extracted with boiling distilled water for 2 h	Zhao, Zhao, Hu [14, 26, 27]
3	Mannose	C_6_H_12_O_6_	*Polygonatum cyrtonema*	Extracted with petroleum ether under reflux for 1.5 h at 75 ℃, followed by 75% ethanol under reflux for 1 h at 85 ℃	Hu [27]
4	Rhamnose	C_6_H_12_O_5_	*Polygonatum*	Extracted with petroleum ether under reflux for 1 h, soaked in 85% ethanol at room temperature for 12 h, and then extracted with boiling distilled water for three times, 2 h each	Zhao, Hu [26, 27]
5	Galactose	C_6_H_12_O_6_	*Polygonatum*	Extracted with petroleum ether under reflux for 1 h, soaked in 85% ethanol at room temperature for 12 h, and then extracted with boiling distilled water for three times, 2 h each	Zhao, Hu [26, 27]
6	Ribose	C_5_H_12_O_5_	*Polygonatum cyrtonema*	Extracted with petroleum ether under reflux for 1.5 h at 75 ℃, followed by 75% ethanol under reflux for 1 h at 85 ℃	Hu [27]
7	Arabinose	C_5_H_10_O_5_	*Polygonatum*	Extracted with petroleum ether under reflux for 1 h, soaked in 85% ethanol at room temperature for 12 h, and then extracted with boiling distilled water for three times, 2 h each	Zhao, Hu [26, 27]
8	Galacturonic acid	C_6_H_10_O_7_	*Polygonatum*	Extracted with petroleum ether under reflux for 1 h, soaked in 85% ethanol at room temperature for 12 h, and then extracted with boiling distilled water for three times, 2 h each	Zhao [26]
9	Xylose	C_5_H_10_O_5_	*Polygonatum*	Extracted with petroleum ether under reflux for 1 h, soaked in 85% ethanol at room temperature for 12 h, and then extracted with boiling distilled water for three times, 2 h each	Zhao [26]

Fructose, with the chemical formula C_6_H_12_O_6_, is the predominant monosaccharide found in *Polygonatum*. It is an isomer of glucose but exhibits a faster metabolic rate [28]. The liver is considered the primary site of fructose metabolism, although Gonzalez et al. suggested that the intestine may also play a significant role in fructose metabolism [29]. Fructose metabolism is closely associated with adipogenesis, and its metabolites can activate adipogenic transcription factors [30]. Mannose, with the chemical formula C_6_H_12_O_6_, is abundant in the fluids and tissues of the human body. Mannose is absorbed and metabolized by the intestines through glycolysis and the tricarboxylic acid cycle [31–33]. Glucose, with the chemical formula C_6_H_12_O_6_, is the most easily absorbed monosaccharide by the body and serves as a direct source of energy [34]. It can easily cross the blood–brain barrier and provides energy to the brain, playing a crucial role in various biological processes [35].

The polysaccharides in *Polygonatum* have anti-inflammatory, antioxidant, and antitumor effects [36, 37], among which glucose has an antagonistic effect on cellular reactive oxygen species levels [38].

### Flavonoids

Flavonoid compounds are secondary metabolites of *Polygonatum*. Scientists have extracted several classes of flavonoid compounds from *Polygonatum*, which are categorized according to their chemical structures into the classes of homoisoflavones, isoflavones, flavonoids, chalcones, and dihydroflavonoids.

Wang et al. isolated 15 flavonoids from the rhizomes of *Polygonatum cyrtonema* by repeated column chromatography and preparative high-performance liquid chromatography techniques, including homoisoflavones and dihydroflavonoids [39]. In another study, Yu et al. employed high-performance liquid chromatography mass spectrometry (HPLC–MS) to isolate seven flavonoids from *Polygonatum sibiricum*, which mainly consisted of homoisoflavones, isoflavones, and flavonoids [40]. Additionally, Jiang et al. further identified four homoisoflavones, two dihydroflavones, two chalcones, and four isoflavones from *Polygonatum sibiricum* and *Polygonatum kingianum* [41]. The flavonoid compounds found in *Polygonatum* are listed in Table 2.

**Table 2:** The flavonoids isolated from *Polygonatum*

NO	Chemical ingredients	Chemical formula	Source	Extraction methods	References
Homoisoflavones					
1	4ʹ,5,7-Trihydroxy-6-methyl-8-methoxy-homoisoflavanon	C_18_H_18_O_6_	*Polygonatum sibiricum*	Extracted with 70% alcohol solution under reflux for three times, 1 h each	Yu [40]
2	4ʹ,7-Dihydroxy-3ʹ-methoxy-homoisoflavanon	C_16_H_12_O_5_	*Polygonatum sibiricum*	Extracted with 70% alcohol solution under reflux for three times, 1 h each	Yu [40]
3	Disporopsin	C_16_H_14_O_6_	*Polygonatum sibiricum*	Extracted with 50% ethanol for three times	Yu [65]
4	(3R)-5,7-Dihydroxy-8-methyl-3-(2′-hydroxy-4ʹ-methoxybenzyl)-chroman-4-one	C_18_H_18_O_6_	*Polygonatum cyrtonema*	Extracted with 95% ethanol	Gan [66]
5	Polygonatone H [(3R)-5,7-Dihydroxy-6-methyl-3-(2ʹ-hydroxy-4ʹ-methoxybenzyl)-chroman-4-on]	C_18_H_18_O_6_	*Polygonatum cyrtonema*	Extracted with 95% ethanol at room temperature for five times	Wang [39]
6	5,7-Dihydroxy-6,8-dimethyl-3-(4ʹ-hydroxybenzyl)-chroman-4-one	C_18_H_18_O_5_	*Polygonatum cyrtonema*	Extracted with 95% ethanol at room temperature for five times	Wang [39]
7	5,7-Dihydroxy-6,8-dimethyl-3-(2ʹ-methoxy-4′-hydroxybenzyl)-chroman-4-one	C_19_H_19_O_6_	*Polygonatum cyrtonema*	Extracted with 95% ethanol at room temperature for five times	Wang [39]
8	5,7-Dihydroxy-6-methyl-3-(4ʹ-hydroxybenzyl)-chroman-4-one	C_17_H_16_O_5_	*Polygonatum cyrtonema*	Extracted with 95% ethanol at room temperature for five times	Wang [39]
9	5,7-Dihydroxy-8-methyl-3-(4ʹ-hydroxybenzyl)-chroman-4-one	C_17_H_16_O_5_	*Polygonatum cyrtonema*	Extracted with 95% ethanol at room temperature for five times	Wang [39]
10	5,7-Dihydroxy-6-methyl-3-(4ʹ-methoxybenzyl)-chroman-4-one	C_19_H_18_O_5_	*Polygonatum cyrtonema*	Extracted with 95% ethanol at room temperature for five times	Wang [39]
11	5,7-Dihydroxy-6,8-dimethyl-3-(4ʹ-methoxybenzyl)-chroman-4-one	C_19_H_20_O_5_	*Polygonatum cyrtonema*	Extracted with 95% ethanol at room temperature for five times	Wang [39]
12	5,7-Dihydroxy-3-(4ʹ-hydroxybenzyl)-chroman-4-one	C_16_H_14_O_5_	*Polygonatum cyrtonema*	Extracted with 95% ethanol at room temperature for five times	Wang [39]
13	5,7-Dihydroxy-6-methyl-3-(2ʹ,4ʹ-dihydroxybenzyl)-chroman-4-one	C_17_H_16_O_6_	*Polygonatum cyrtonema*	Extracted with 95% ethanol at room temperature for five times	Wang [39]
14	5,7-Dihydroxy-3-(2ʹ-hydroxy-4ʹ-methoxybenzyl)-chroman-4-one	C_17_H_16_O_6_	*Polygonatum cyrtonema*	Extracted with 95% ethanol at room temperature for five times	Wang [39]
Isoflavone					
15	Tectoridin	C_22_H_22_O_11_	*Polygonatum sibiricum*	Extracted with industrial alcohol for five times, 3 d each	Jiang [41]
16	2′,7-Dihydroxy-3′,4′-dimethoxyisoflavan	C_17_H_18_O_5_	*Polygonatum kingianum*	Extracted with industrial alcohol for five times, 3 d each	Jiang [41]
17	2′,7-Dihydroxy-3′,4′-dimethoxyisoflavanoside	C_23_H_28_O_10_	*Polygonatum kingianum*	Extracted with industrial alcohol for five times, 3 d each	Jiang [41]
18	4′,7-Dihydroxy-3′-methoxyisoflavone	C_16_H_12_O_5_	*Polygonatum kingianum*	Extracted with industrial alcohol for five times, 3 d each	Jiang [41]
Flavones					
19	Apigenin	C_15_H_10_O_5_	*Polygonatum cyrtonema*	Extracted with methanol	Xu [67]
20	Apigenin-7-O-β-D-glucoside	C_21_H_20_O_10_	*Polygonatum sibiricum*	Extracted with 75% ethanol	Ren [68]
21	Apigenin-8-c-galactoside	C_21_H_20_O_10_	*Polygonatum sibiricum*	Extracted with 70% alcohol solution under reflux for three times, 1 h each	Yu [40]
22	Liquiritigenin	C_15_H_12_O_4_	*Polygonatum kingianum*	Extracted with 95% ethanol under reflux condition	Wang [69]
23	Neoliquiritin	C_21_H_22_O_9_	*Polygonatum kingianum*	Extracted with 95% ethanol under reflux condition	Wang [69]
24	Kaempferol	C_15_H_10_O_6_	*Polygonatum sibiricum*	Extracted with three times volume of methanol at 60 ℃ for 24 h	Park [44]
25	Myricetin	C_15_H_10_O_8_	*Polygonatum sibiricum*	Extracted with 75% ethanol	Gao [70]
26	Rutin	C_27_H_30_O_16_	*Polygonatum sibiricum*	Extracted with 70% methanol	Wang [19]
27	Kaempferol-3-O-(2″-O-β-D-glucopyranosyl)-β-D-glucopyranoside	C_28_H_24_O_15_	*Polygonatum sibiricum*	Extracted with 70% ethanol at 80 ℃ for three times, 2 h each	Wang [71]
28	Isorhamnetin	C_16_H_12_O_7_	*Polygonatum sibiricum*	Extracted with 70% methanol	Wang [19]
29	Quercetin	C_15_H_10_O_7_	*Polygonatum sibiricum*	Extracted with cellulase in 90% ethanol under ultrasonic condition	Guo [43]
30	Baicalein	C_15_H_10_O_5_	*Polygonatum sibiricum*	Extracted with cellulase in 90% ethanol under ultrasonic condition	Guo [43]
Chalcones					
31	Isoliquiritigenin	C_15_H_12_O_4_	*Polygonatum kingianum*	Extracted with 70% ethanol under reflux condition	Li [72]
32	Neoisoliquiritin	C_21_H_22_O_9_	*Polygonatum kingianum*	Extracted with 70% ethanol under reflux condition	Li [72]
33	Polygonatone D (2′,4′,4-trihydroxyl-3′-methyl-6′-methoxyldihydrochalcone)	C_17_H_18_O_5_	*Polygonatum cyrtonema*	Extracted with 95% ethanol at room temperature for five times	Wang [39]
Dihydroflavones					
34	(3S)-3,7-Dihydroxy-8-methoxy-3-(3′,4′-methylenedioxybenzyl)-chroman-4-one	C_18_H_16_O_7_	*Polygonatum cyrtonema*	Extracted with methanol	Xu [67]
35	7-Hydroxy-3-(2′-hydroxy-3′,4′-dimethoxybenzyl)-chroman-4-one	C_17_H_18_O_5_	*Polygonatum kingianum*	Extracted with 95% ethanol under reflux condition	Wang [69]
36	7-Hydroxy-3-(3′-methoxy-4′-hydroxybenzyl)-chroman-4-one	C_16_H_12_O_5_	*Polygonatum kingianum*	Extracted with 70% ethanol under reflux condition	Chen [73]
37	5-Hydroxy-7-methoxyl-3-(2′-hydroxy-4′-methoxybenzyl)-chroman-4-one	C_18_H_18_O_6_	*Polygonatum sibiricum*	Extracted with 95% ethanol under reflux for three times, 2 h each	Chen [74]
38	Odoratumone A	C_19_H_20_O_6_	*Polygonatum sibiricum*	Extracted with 70% alcohol solution under reflux for three times, 1 h each	Yu [40]
39	5,7-Dihydroxy-6-methyl-8-methoxy-3-(4′-hydroxybenzyl)-chroman-4-one	C_18_H_18_O_6_	*Polygonatum sibiricum*	Extracted with 70% alcohol solution under reflux for three times, 1 h each	Yu [40]
40	5,7-Dihydroxy-3-(4′-methoxybenzyl)-chroman-4-one	C_17_H_16_O_5_	*Polygonatum cyrtonema*	Extracted with 70% ethanol for five times	Wang [75]
41	5-Hydroxy-7-methoxy-6,8-dimethyl-3-(2′-hydroxy-4′-methoxybenzyl)-chroman-4-one	C_20_H_22_O_6_	*Polygonatum cyrtonema*	Extracted with 70% ethanol for five times	Wang [75]
42	5,7-Dihydroxy-6,8-dimethyl-3-(2′,4′-dihydroxybenzyl)-chroman-4-one	C_18_H_18_O_6_	*Polygonatum sibiricum*	Extracted with 75% ethanol	Ren [68]
43	5,7-Dihydroxy-3-(4′-hydroxybenzylidene)-chroman-4-one	C_16_H_12_O_5_	*Polygonatum cyrtonema*	Extracted with 70% ethanol for five times	Wang [75]
44	(6aR, 11aR) -10-Hydroxy-3,9-dimethoxypterocarpan	C_17_H_16_O_5_	*Polygonatum kingianum*	Extracted with 70% ethanol under reflux condition	Li-2008[72]
45	Isomucronulatol	C_17_H_18_O_5_	*Polygonatum kingianum*	Extracted with 95% ethanol under reflux condition	Wang-2003[69]

*Polygonatum* is also rich in other flavonoids, such as rutin, quercetin, isorhamnetin, kaempferol, and baicalein [19, 42–44]. Upon ingestion, rutin is hydrolyzed in the body and converted to quercetin, which is further metabolized to isorhamnetin after absorption in the intestines [45]. Quercetin undergoes hepatic metabolism to form glucuronic acid, methyl, and sulfate conjugates, which are eventually excreted through the kidneys [46]. Isorhamnetin, predominantly excreted through the kidneys, is found in the bloodstream and urine in the conjugated form with glucuronic acid [47]. Kaempferol is metabolized in the liver as glucuronic acid, methyl, and sulfate metabolites [46]. Baicalein undergoes methylation in the human body and is metabolized to oroxylin A, which is then converted to the final metabolites: baicalin, oroxylin A-7-O-b-D-glucuronide, and 5,7-dihydroxy-6-O-b-D-glucuronide, and is finally excreted in the urine [48].

Flavonoid compounds found in *Polygonatum* exhibit various beneficial effects, including antitumor, antihyperglycemic, anti-inflammatory, and antioxidant properties [49–63]. Specifically, kaempferol has been shown to decrease the expression of transcription factors involved in lipid formation [44], while baicalein enhances the activity of the Adenosine 5ʹ-monophosphate (AMP)-activated protein kinase/nuclear factor erythroid 2-related factor/heme oxygenase-1 (AMPK/Nrf2/HO-1) signaling pathway, thereby inhibiting ferroptosis in chondrocytes [64].

### Saponins

The saponin compounds found in *Polygonatum* can be divided into two categories: steroidal saponins and triterpenoid saponins. A total of 86 steroidal saponins and 12 triterpenoid saponins have been identified and isolated from *Polygonatum*. The specific saponin compounds are listed in Table 3.

**Table 3:** The saponins isolated from *Polygonatum*

NO	Chemical ingredients	Chemical formula	Source	Extraction methods	References
Steroid saponins					
1	Sibiricoside A	C_57_H_94_O_28_	*Polygonatum sibiricum*	Extracted with methanol at room temperature for three times, 12 h each	Son [77]
2	Protodioscin	C_51_H_84_O_22_	*Polygonatum sibiricum*	Extracted with 75% ethanol	Ren [68]
3	Protogracillin	C_51_H_84_O_23_	*Polygonatum sibiricum*	Extracted with 75% methanol for three times, 24 h each	Ren [68]
4	Methyl protodioscin	C_52_H_86_O_22_	*Polygonatum sibiricum*	Extracted with 75% methanol for three times, 24 h each	Ren [68]
5	Methyl protogracillin	C_52_H_86_O_23_	*Polygonatum sibiricum*	Extracted with 75% methanol for three times, 24 h each	Ren [68]
6	Polygonoide A	C_57_H_92_O_27_	*Polygonatum sibiricum*	Extracted with 75% methanol for three times, 24 h each	Ren [68]
7	Polygonoide B	C_43_H_68_O_19_	*Polygonatum sibiricum*	Extracted with methanol at room temperature for three times, 12 h each	Son [77]
8	Kingianoside Z	C_57_H_90_O_29_	*Polygonatum sibiricum*	Extracted with 95% ethanol at room temperature for three times	Zhang [89]
9	(25RS)-26-(β-glucopyranosyl)-22-Methylfurost-5-ene-3β,14α,26-triol 3-O-β-lycotetraoside	C_57_H_94_O_29_	*Polygonatum sibiricum*	Extracted with methanol at room temperature for three times, 12 h each	Son [77]
10	(25RS)-Kingianoside C	C_45_H_72_O_20_	*Polygonatum kingianum*	Extracted with 50% ethanol for three times	Zhang [76]
11	(25RS)-Kingianoside E	C_51_H_82_O_25_	*Polygonatum kingianum*	Extracted with 50% ethanol for three times	Zhang [76]
12	(25RS)-Kingianoside F	C_57_H_94_O_29_	*Polygonatum sibiricum*	Extracted with methanol at room temperature for three times, 12 h each	Son [77]
13	Sibiricogenin 3-O-β-Lycotetraoside	C_57_H_94_O_29_	*Polygonatum sibiricum*	Extracted with methanol at room temperature for three times, 12 h each	Son [77]
14	(25RS, 22ξ)-Hydroxylwattinoside C	C_45_H_74_O_20_	*Polygonatum kingianum*	Extracted with 50% ethanol for three times	Zhang [76]
15	22-Hydroxylwattinoside C	C_45_H_74_O_20_	*Polygonatum kingianum*	Extracted with 50% ethanol for three times	Zhang [76]
16	(25R,22)-Hydroxylwattinoside C	C_45_H_74_O_20_	*Polygonatum kingianum*	Extracted with 50% ethanol for three times	Zhang [76]
17	(25RS)-Kingianoside D	C_45_H_72_O_19_	*Polygonatum kingianum*	Extracted with methanol under heating condition	Li [90]
18	Huangjinoside A	C_33_H_50_O_8_	*Polygonatum sibiricum*	Extracted with water followed by chloroform	Ren [68]
19	Huangjinoside B	C_39_H_60_O_15_	*Polygonatum sibiricum*	Extracted with 70% alcohol solution under reflux for three times, 1 h each	Ren [68]
20	Huangjinoside C	C_32_H_52_O_9_	*Polygonatum sibiricum*	Extracted with water followed by chloroform	Ren [68]
21	Huangjinoside D	C_33_H_52_O_9_	*Polygonatum sibiricum*	Extracted with water followed by chloroform	Ren [68]
22	Huangjinoside E	C_39_H_62_O_14_	*Polygonatum sibiricum*	Extracted with water followed by chloroform	Ren [68]
23	Huangjinoside F	C_33_H_62_O_15_	*Polygonatum sibiricum*	Extracted with water followed by chloroform	Ren [68]
24	Huangjinoside G	C_45_H_72_O_19_	*Polygonatum sibiricum*	Extracted with water followed by chloroform	Ren [68]
25	Huangjinoside H	C_45_H_72_O_20_	*Polygonatum sibiricum*	Extracted with water followed by chloroform	Ren [68]
26	Huangjinoside I	C_38_H_60_O_15_	*Polygonatum sibiricum*	Extracted with water followed by chloroform	Ren [68]
27	Huangjinoside J	C_39_H_62_O_15_	*Polygonatum sibiricum*	Extracted with water followed by chloroform	Ren [68]
28	Huangjinoside K	C_39_H_62_O_16_	*Polygonatum sibiricum*	Extracted with water followed by chloroform	Ren [68]
29	Huangjinoside L	C_39_H_62_O_15_	*Polygonatum sibiricum*	Extracted with water followed by chloroform	Ren [68]
30	Huangjinoside M	C_39_H_62_O_16_	*Polygonatum sibiricum*	Extracted with water followed by chloroform	Ren [68]
31	Huangjinoside N	C_45_H_72_O_21_	*Polygonatum sibiricum*	Extracted with water followed by chloroform	Ren [68]
32	Huangjinoside O	C_45_H_72_O_20_	*Polygonatum sibiricum*	Extracted with water followed by chloroform	Ren [68]
33	Neosibiricoside A	C_47_H_74_O_21_	*Polygonatum sibiricum*	Extracted with methanol	Ahn [91]
34	Neosibiricoside B	C_52_H_82_O_24_	*Polygonatum sibiricum*	Extracted with methanol	Ahn [91]
35	Neosibiricoside C	C_52_H_82_O_23_	*Polygonatum sibiricum*	Extracted with methanol	Ahn [91]
36	Neosibiricoside D	C_45_H_72_O_18_	*Polygonatum sibiricum*	Extracted with methanol	Ahn [91]
37	(25RS)-Kingianoside A	C_39_H_60_O_14_	*Polygonatum kingianum*	Extracted with 50% ethanol for three times	Yu [65]
38	Kingianoside B	C_39_H_60_O_13_	*Polygonatum kingianum*	Extracted with methanol under heating condition	Li [90]
39	(25R)-Kingianoside G	C_45_H_70_O_20_	*Polygonatum kingianum*	Extracted with 50% ethanol for three times	Yu [65]
40	Kingianoside H	C_39_H_60_O_15_	*Polygonatum kingianum*	Extracted with 45% acetone for three times	Yu [92]
41	Kingianoside I	C_45_H_70_O_20_	*Polygonatum kingianum*	Extracted with 45% acetone for three times	Yu [92]
42	Kingianoside K	C_44_H_68_O_17_	*Polygonatum kingianum*	Extracted with 45% acetone for three times	Yu [79]
43	Cyrtonemoside A	C_51_H_80_O_24_	*Polygonatum cyrtonema*	Extracted with 80% ethanol for four times	Ma [93]
44	Pratioside D_1_	C_45_H_70_O_19_	*Polygonatum kingianum*	Extracted with 45% acetone for three times	Yu [79]
45	(25RS)-Pratioside D1	C_45_H_70_O_19_	*Polygonatum kingianum*	Extracted with 45% acetone for three times	Yu [79]
46	Sibiricoside B	C_50_H_80_O_24_	*Polygonatum sibiricum*	Extracted with methanol at room temperature for three times, 12 h each	Son [77]
47	Neoprazerigenin 3-O-β- lycotetraoside	C_50_H_80_O_23_	*Polygonatum sibiricum*	Extracted with methanol at room temperature for three times, 12 h each	Son [77]
48	Polygonatoside C_1_	C_39_H_60_O_15_	*Polygonatum kingianum*	Extracted with 45% acetone for three times	Yu [79]
49	(25S)-Pratioside D_1_	C_45_H_70_O_19_	*Polygonatum sibiricum*	Extracted with methanol at room temperature for three times, 12 h each	Son [77]
50	(25R)-Pratioside D_1_	C_45_H_70_O_19_	*Polygonatum kingianum*	Extracted with 50% ethanol for three times	Yu [65]
51	(25RS)-Neobotogenin	C_27_H_40_O_4_	*Polygonatum cyrtonema*	Extracted with 80% ethanol for four times	Ma [93]
52	Disporopsin	C_16_H_14_O_6_	*Polygonatum kingianum*	Extracted with 50% ethanol for three times	Yu [65]
53	Saponin Tg	C_51_H_82_O_21_	*Polygonatum kingianum*	Extracted with 50% ethanol for three times	Yu [65]
54	Saponin Pb	C_51_H_82_O_20_	*Polygonatum kingianum*	Extracted with 45% acetone for three times	Yu [79]
55	Dioscin	C_45_H_72_O_17_	*Polygonatum kingianum*	Extracted with 45% acetone for three times	Yu [79]
56	Gracillin	C_54_H_92_O_13_	*Polygonatum kingianum*	Extracted with 45% acetone for three times	Yu [79]
57	Saponin Pa	C_44_H_70_O_16_	*Polygonatum kingianum*	Extracted with 45% acetone for three times	Yu [79]
58	Funkioside C	C_39_H_62_O_13_	*Polygonatum kingianum*	Extracted with methanol under heating condition	Li [90]
59	Saponin Tb	C_39_H_62_O_13_	*Polygonatum kingianum*	Extracted with 45% acetone for three times	Yu [79]
60	Daucosterol	C_35_H_60_O_6_	*Polygonatum kingianum*	Extracted with 45% acetone for three times	Yu [79]
61	Ginsenoside Rb_1_	C_56_H_90_O_24_	*Polygonatum kingianum*	Extracted with 45% acetone for three times	Yu [79]
62	Diosgenin	C_27_H_42_O_3_	*Polygonatum cyrtonema*	Extracted with 95% ethanol for two times, 4 h each	Zeng [94]
63	Trillin	C_33_H_52_O_8_	*Polygonatum sibiricum*	Extracted with 75% ethanol for three times, 24 h each	Ren [68]
64	Huangjingenin	C_27_H_42_O_5_	*Polygonatum sibiricum*	Extracted with water followed by chloroform	Ren [68]
65	(25S) Spirost-5-en-12-one-3-O-β-D-glucopyranosyl-(1→2)- [β-D-xylopyranosyl-(1→3)]-β-D-glucopyranosyl-(1→4)-β- D-galactopyranoside	C_50_H_78_O_23_	*Polygonatum cyrtonema*	Extracted with 80% ethanol for four times	Ma [93]
66	(3β, 25RS)-Spirost-5-en-12-one-3-[(O-β-D-glucopyranosyl-(12)-O-[β-D-glucopyranosyl-(1→3)]-O-β-D-xylopyranosyl-(1→4)-β-D-galactopyranosyl)-oxy	C_50_H_78_O_23_	*Polygonatum cyrtonema*	Extracted with 80% ethanol for four times	Ma [93]
67	(25RS)-Spirostan-5-en-12-one-3-O-D-glucopyranosyl-(1→2)-O- [β-D-xylopyranosyl (1→3)] -O-β-D-glucopyranosyl (1→4)-β-D-galactopyranoside	C_50_H_78_O_23_	*Polygonatum cyrtonema*	Extracted with 80% ethanol for four times	Ma [93]
68	(25R)-Spirost-5-en-3β,17α-diol-3-O-β-D-glucopyranosyl-(1→3)-[α-L-rhamnopyranosyl-(1→2)]-β-D-glucopyranosid	C_45_H_72_O_18_	*Polygonatum kingianum*	Extracted with 45% acetone for three times	Yu [92]
69	(25R)-Spirost-5-en-3β,17α-diol-3-O-β-D-glucopyranosyl (1→2)-β-D-glucopyranosyl (1→4)-β-D-fucopyranosyl	C_45_H_72_O_19_	*Polygonatum sibiricum*	Extracted with 70% ethanol under reflux for three times	Ren [68]
70	O-β-D-Glucopyranosyl-(1→2)-β-D-glucopyranosyl (1→4)-β-D-fucopyranosyl	C_45_H_72_O_18_	*Polygonatum sibiricum*	Extracted with 70% ethanol under reflux for three times	Ren [68]
71	(25R) -Spirost-5-en-3β, 17α-diol-3-O-β-D-glucopyranosyl (1→4) -β-D-galactopyranoside	C_39_H_62_O_14_	*Polygonatum sibiricum*	Extracted with 70% ethanol under reflux for three times	Ren [68]
72	(25RS)-Spirost-5-en-3β,17α-diol-3-O-β-D-glucopyranosyl (1→4)-β-D-fucopyranosyl	C_39_H_62_O_13_	*Polygonatum sibiricum*	Extracted with 70% ethanol under heating condition for three times	Tang [95]
73	(25RS)-Spirost-5-en-3β,12β-diol-3-O-β-D-glucopyranosyl (1→4)-β-D- fucopyranosyl	C_39_H_62_O_13_	*Polygonatum sibiricum*	Extracted with 70% ethanol under heating condition for three times	Tang [95]
74	(23S, 25R)-Spirost-5-ene-3β,14α,23-ttiol	C_27_H_42_O_5_	*Polygonatum sibiricum*	Extracted with methanol at room temperature for three times, 12 h each	Son [77]
75	3-O-β-D-Glucopyranosyl (1→3)-β-D-glucopyranosyl (1 →4)- [α-L-rhamnopyranosyl (1→2)]-β-D-glucopyranoside-diosgeni	C_51_H_82_O_22_	*Polygonatum sibiricum*	Extracted with 80% ethanol for three times, 2 h each	Xu [96]
76	3-O-β-D-Glucopyranosyl (1→4)- [α-L-rhamnopyranosyl (1→2)]-β-D-glucopyranosyl-diosgenin (PO-3)	C_45_H_72_O_17_	*Polygonatum sibiricum*	Extracted with 80% ethanol for three times, 2 h each	Xu [96]
77	3-O-β-D-α-L-Rhamnopyranosyl (1→4)- [α-L-rhamnopyranosyl (1→2)]-β-D-glucopy-ranoside-diosgenin	C_45_H_72_O_16_	*Polygonatum sibiricum*	Extracted with 80% ethanol for three times, 2 h each	Xu [96]
78	3-O-β-D-Glucopyranosyl (1→4)- [α-L-rhamnopyranosyl (1→2)]-β-D-glucopyranoside-diosgenin	C_45_H_72_O_17_	*Polygonatum sibiricum*	Extracted with 80% ethanol for three times, 2 h each	Xu [96]
79	(25RS) -Spirost-5-en-3β, 12β-diol-3-O-β-D-glucopyranosyl (1→4) -β-D-fucosopyranoside	C_39_H_62_O_13_	*Polygonatum sibiricum*	Extracted with 70% ethanol under heating condition for three times, 2 h each	Tang [95]
80	(25RS)3-β-Hydroxyspirost-5-en-12-one	C_27_H_40_O_4_	*Polygonatum cyrtonema*	Extracted with 80% ethanol for four times	Ma [93]
81	(3β, 25R)-26-(β-D-Glucopyranosyloxy)-22-hydroxyfurost-5-en-3-yl 4-O-β-D-glucopyranosyl-β-D-galactopyranoside	C_45_H_72_O_19_	*Polygonatum kingianum*	Extracted with methanol under heating condition	Li [90]
82	(3β, 25R)-26-(β-D-Glucopyranosyloxy)-22-methoxyfurost-5-en-3-yl 4-O-β-D- glucopyranosyl	C_46_H_76_O_19_	*Polygonatum kingianum*	Extracted with methanol under heating condition	Li [90]
83	26-O-β-D-Glucopyranose-3β, 26-diol-(25R)-Δ5, 22 (23) -diene-furost-3-O-β-D-glucopyranosid	C_39_H_62_O_13_	*Polygonatum sibiricum*	Extracted with 75% ethanol for three times, 24 h each	Ren [68]
84	26-O-β-D-Glucopyranose-3β, 26-diol-(25R)-Δ5, 20 (22) -diene-furanost-3-O-β-D-glucopyranoside	C_39_H_62_O_13_	*Polygonatum sibiricum*	Extracted with 75% ethanol for three times, 24 h each	Ren [68]
85	(3β, 25R)-Furost-5-en-12-one,3-[(4-O-β-D-glucopyranosyl-β-D-galactopyranosyl) oxy]-26-(β-D-glucopyranosyloxy)-22-methoxy	C_46_H_74_O_20_	*Polygonatum kingianum*	Extracted with methanol under heating condition	Li [90]
86	(3β, 25R)-Furost-5-en-12-one,3-[(6- deoxy-4-O-β-D- galactopyranosyl) oxy] -26-(β-D- glucopyranosyloxy)-22-methoxy	C_46_H_74_O_19_	*Polygonatum kingianum*	Extracted with methanol under heating condition	Li [90]
Triterpenoid saponins					
87	Ginsenoside Rb_1_	C_54_H_92_O_13_	*Polygonatum kingianum*	Extracted with 45% acetone for three times	Yu [79]
88	Ginsenoside Rc	C_53_H_90_O_22_	*Polygonatum kingianum*	Extracted with 50% ethanol for three times	Yu [65]
89	Pseudoginsenoside F_11_	C_42_H_72_O_14_	*Polygonatum sibiricum*	Extracted with 50% ethanol under reflux for three times	Ren [68]
90	Polygonoide C	C_48_H_78_O_19_	*Polygonatum sibiricum*	Extracted with 20% ethanol at 60 ℃ for three times, 2 h each	Hu [97]
91	Polygonoide D	C_49_H_80_O_19_	*Polygonatum sibiricum*	Extracted with 20% ethanol at 60 ℃ for three times, 2 h each	Hu [97]
92	Polygonoide E	C_72_H_118_O_39_	*Polygonatum sibiricum*	Extracted with 20% ethanol at 60 ℃ for three times, 2 h each	Hu [97]
93	Madecassoside	C_48_H_78_O_20_	*Polygonatum sibiricum*	Extracted with 70% ethanol at 60 ℃ for three times, 2 h each	Ren [68]
94	Asiaticoside	C_48_H_78_O_19_	*Polygonatum sibiricum*	Extracted with 70% ethanol at 60 ℃ for three times, 2 h each	Ren [68]
95	3β, 30β-Dihydroxy- (3→1) glucose-(2→1) glucose-oleanane	C_42_H_72_O_12_	*Polygonatum sibiricum*	Extracted with 70% ethanol for three times	Ren [68]
96	3β-Hydroxy- (3→1) glucose- (4→1) glucose-oleanane	C_42_H_72_O_11_	*Polygonatum sibiricum*	Extracted with 70% ethanol for three times	Ren [68]
97	3β-Hydroxy- (3→1) glucose- (4→1) glucose- (28→1) arabinose- (2→1) arabinose-oleanolic acid	C_52_H_84_O_21_	*Polygonatum sibiricum*	Extracted with 70% ethanol for three times	Ren [68]
98	3β-Hydroxy- (3→1) glucose- (2→1) glucose-oleanolic acid	C_42_H_68_O_13_	*Polygonatum sibiricum*	Extracted with 70% ethanol for three times	Ren [68]

Zhang et al. conducted ethanol extraction and isolated ten kinds of steroidal saponins from *Polygonatum kingianum* [76]. Song et al. isolated nine saponins, all steroidal saponins, from *Polygonatum sibiricum* by methanol extraction [77]. Among these, sibiricoside A is a specific steroidal saponin that is predominantly distributed in the stomach, small intestine, kidney, liver, and other tissues of rats, and is mainly excreted through feces [78]. Yu et al. isolated 13 saponins from *Polygonatum kingianum* by acetone extraction [79]. Among which, ginsenoside Rb1 exhibits anti-inflammatory, antioxidant, and anti-apoptotic effects [80, 81], and is absorbed in the human gut after oral administration, undergoing metabolism by intestinal flora to produce more active metabolites [82]. Methanol and ethanol have strong polarity, which are easier to extract steroid saponins with the same polarity. On the contrary, acetone with weak polarity, is more suitable for extracting triterpene saponins.

**Fig. 1:**
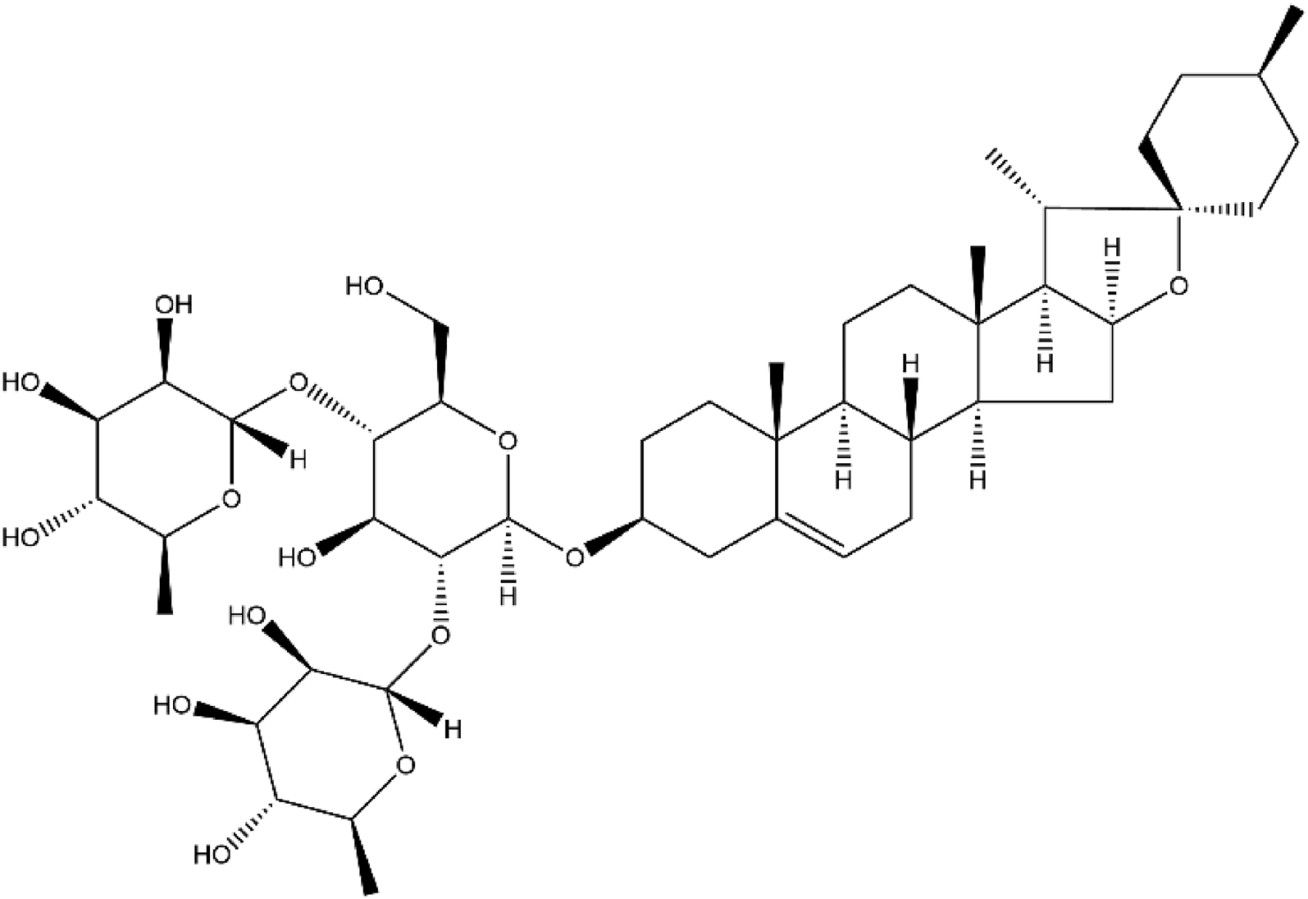
Chemical structures of dioscin

Dioscin, with the chemical formula C_45_H_72_O_16_, is the principal active compound among the class of *Polygonatum* saponins and has been extensively studied. Its structural formula is shown in Fig. 1. Dioscin has limited bioavailability, and it was discovered to be absorbed slowly in the intestinal tract of rats, with only a small amount being metabolized into diosgeninogen and primarily excreted in feces. Dioscin has been found to possess anti-inflammatory, antioxidant, and antitumor properties [83–87], especially has effectiveness in inhibiting adriamycin-induced oxidative damage in the myocardium [88].

### Others

**Fig. 2:**
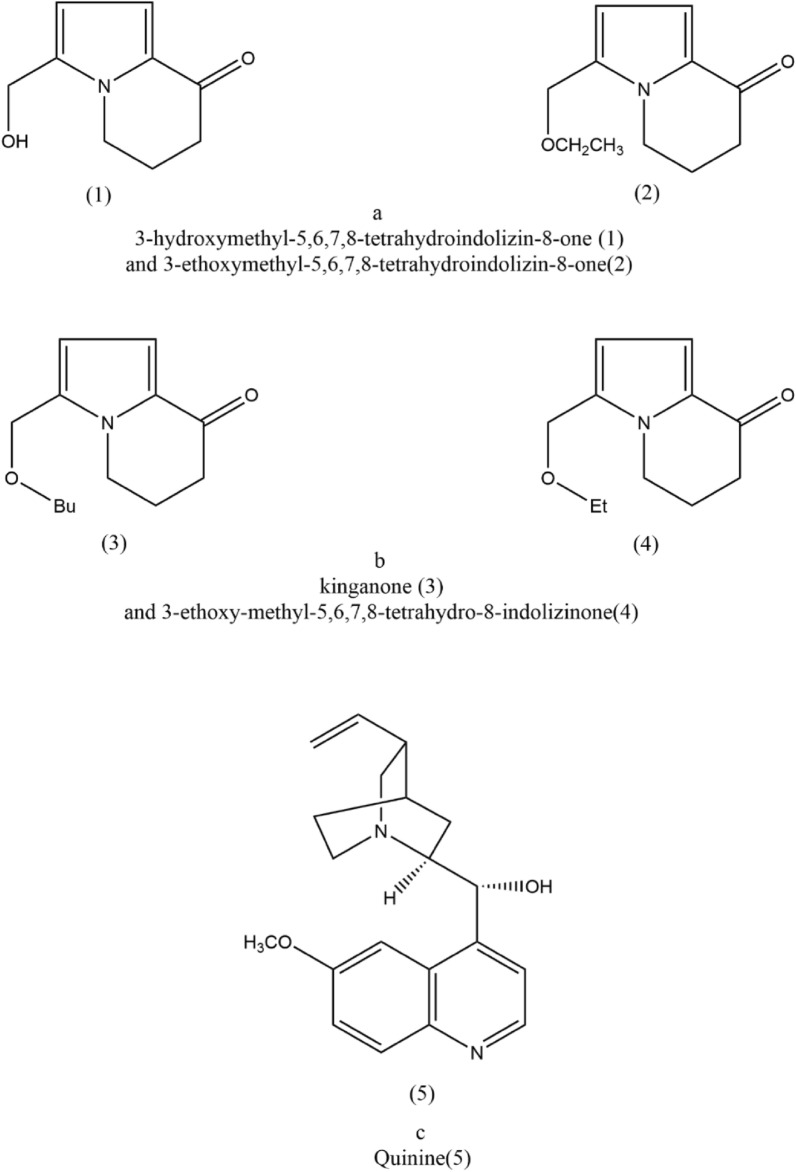
Chemical structures of alkaloid

*Polygonatum* contains trace amounts of alkaloids, and 18 alkaloids have been identified. Based on their chemical structures, these alkaloids can be categorized as amides, indolizines, phenylpropanoids, quinolines, and purines [98]. Sun et al. isolated two alkaloids, namely 3-hydroxymethyl-5,6,7,8-tetrahydroindolizin-8-one(1) and 3-ethoxymethyl-5,6,7,8-tetrahydroindolizin-8-one(2) from *Polygonatum* [99], and their specific structures are depicted in Fig. 2a. Wang et al. isolated two alkaloids, kinganone(3) and 3-ethoxy-methyl-5,6,7,8-tetrahydro-8-indolizinone(4) from the rhizomes of *Polygonatum* [100], and their specific structures are illustrated in Fig. 2b. These two alkaloids exhibit weak antibacterial and antifungal activities. Additionally, Virk et al. isolated Quinine(5) from *Polygonatum* [101], a quinoline alkaloid with anti-malarial activity [102–104], and its structure is presented in Fig. 2c.

Lignans are present in minimal amounts in *Polygonatum* and have been identified to include syringaresinols, liriodendrin, pinoresinols, and furanones [98]. Lignans are natural compounds formed by the polymerization of two molecules of phenylpropanoid derivatives. They can be absorbed and metabolized in the intestinal tract, leading to the production of polyphenolic compounds [105]. Lignans have been shown to possess antitumor effects [106–108]. Osmakov et al. found that most lignans exhibit both anti-inflammatory and antioxidant properties [109].

### Application and pharmacological mechanism of *Polygonatum* and its active ingredients in CVDs

**Fig. 3:**
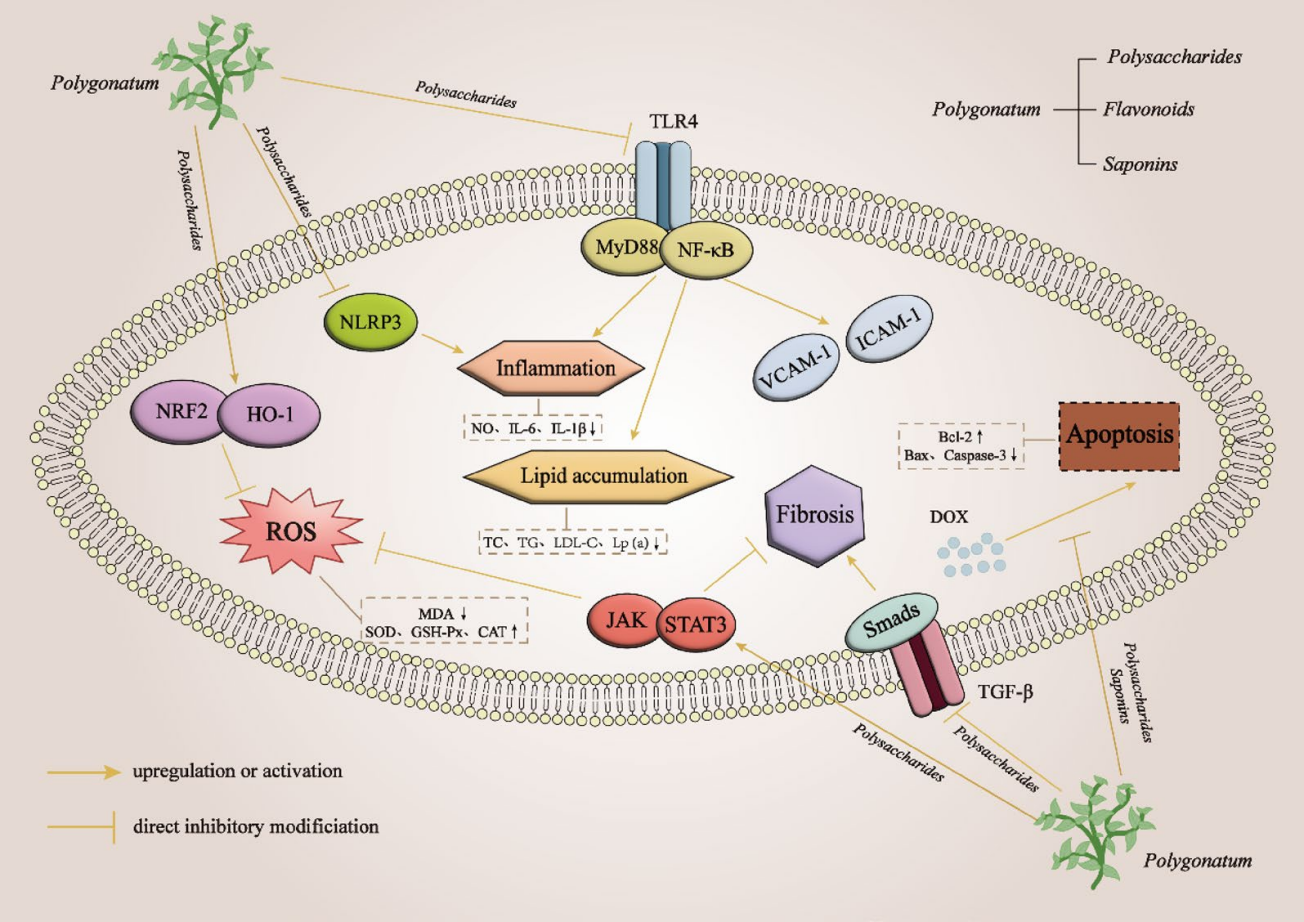
Pharmacological mechanism of *Polygonatum* for prevention and treatment of CVDs Nrf2, nuclear factor erythroid 2-related factor; HO-1, heme oxygenase-1; ROS, reactive oxygen species; NLRP3, nod-like receptor protein 3; TLR4, Toll-like receptor4; MyD88, myeloid differentiation factor88; NF-κB, nuclear factor kappa-B; VCAM-1, vascular cell adhesion molecule-1; ICAM-1, intercellular adhesion molecule-1; TGF-β, transforming growth factor beta; JAK, Janus kinase; STAT3, signal transducer and activator of transcription 3; DOX, doxorubicin; MDA, malondialdehyde; SOD, superoxide dismutase; GSH-Px, glutathione peroxidase; CAT, catalase; NO, nitric oxide; IL-6, interleukin-6; IL-1β, interleukin-1 beta; TC, total cholesterol; TG, triglyceride; LDL-C, low-density lipoprotein cholesterol; Lp(a), lipoprotein; Bcl-2, B-cell lymphoma-2; Bax, BCL2-associated X

The pharmacological mechanisms underlying the preventive and therapeutic effects of *Polygonatum* and its active ingredients in CVDs are multifaceted, primarily encompassing anti-oxidative stress in cardiomyocytes, lipid regulation, anti-atherosclerosis, anti-inflammation, and anti-myocardial fibrosis. Moreover, *Polygonatum* and its active ingredients exhibit distinct advantages in preventing pharmacological cardiotoxicity. The subsequent sections will comprehensively analyze and discuss these aspects (Fig. 3, Table 4).

**Table 4:** Application and pharmacological mechanism of *Polygonatum* and its active ingredients in CVDs

NO	Pharmacological properties	Pharmacological mechanism	Chemical ingredients of *Polygonatum*
1	Anti-oxidative stress	①Scavenging excess free radicals and inhibiting lipid peroxidation ②Regulation of TGF-β1/Smads, JAK/STAT3, NRF2/HO-1 signaling pathways	*Polygonatum sibiricum* polysaccharides/Crude *P. Cyrtonema* and steam-processed *P. cyrtonema* extracted from *Polygonatum cyrtonema*/Flavonoids and saponins extracted from *Polygonatum*
2	Lipid and glucose regulation, and anti-atherosclerosis	①Regulation of miR-340-3p/IRAK3 to reduce lipid accumulation and expression of inflammatory factors ②Inhibition of late glycosylation end-product formation and GLUT2 ③Mediating a miRNA network with miR-484-Bacteroides/Roseburia axis as the pivot to alleviate lipid metabolism disorders	*Polygonatum sibiricum* polysaccharides/Steroid saponins and homoisoflavones extracted from *Polygonatum*/Saponins in *Polygonatum*
3	Anti-inflammation and anti-myocardial fibrosis	①Modulation of TLR4-MyD88-NF-κB signaling pathway reduces inflammatory factor expression ②Regulation of TGF-β1/Smads, JAK/STAT3, NRF2/HO-1 signaling pathways ③Inhibition of NLRP3 inflammasome and NF-κB signaling pathway-mediated inflammatory responses	Compound β-carboline extracted from *Polygonatum sibiricum Red. Led.*/*Polygonatum sibiricum* polysaccharides/The methanolic extract of *Polygonatum*
4	Amelioration of drug cardiotoxicity	①Anti-oxidative stress, anti-inflammatory, and anti-cardiomyocyte apoptosis ②Regulation of NRF2/HO-1and Sirt2 signaling pathways	*Polygonatum sibiricum* polysaccharides/Dioscin extracted from *Polygonatum*

### Anti-oxidative stress

Oxidative stress plays a significant role in the pathogenesis of various cardiovascular diseases [110, 111]. It arises from the excessive accumulation of free radicals or reactive oxygen species (ROS) [112]. Under normal conditions, ROS levels can be kept in balance by a complex antioxidant system. During disease progression, oxidative stress injury is induced when antioxidant enzymes, such as catalase, superoxide dismutase (SOD), and glutathione/glutathione synthase (GSH/GSS), are unable to neutralize the overproduced ROS [113]. These oxygen-free radicals exceed the ability of endothelial cells to protect themselves and, through a variety of mechanisms, lead to endothelial cell dysfunction, a process that occurs in most cardiovascular diseases [110, 114, 115]. In contrast to other tissues, the myocardium is more susceptible to oxidative injury due to its high oxidative metabolism and low antioxidant defenses [116, 117]. Notably, several studies have shown that *Polygonatum* can exert antioxidant effects by mediating multiple oxidative stress signaling pathways, making it a natural antioxidant [118, 119].

#### In vitro study

PSPs were found to have scavenging effects on 2,2-diphenyl-1-picrylhydrazyl (DPPH), hydroxyl radicals, superoxide anion radicals, and special scavenging effects on ferrous iron chelating ability, indicating that PSPs may be a potential antioxidant [118]. Li et al. [12] utilized a hypoxia inducer to execute a cardiomyocyte hypoxia model in rat H9c2 cardiomyocytes and then applied *Polygonatum kingianum*’s effective ingredients, saponins Gracillin and flavonoids Liquiritigenin. The results demonstrated that the intervention of 20 μM Gracillin significantly reduced the expression of malondialdehyde (MDA) and lactate dehydrogenase (LDH), while increasing the expression level of superoxide dismutase (SOD). Similarly, liquiritigenin exhibited similar effects following the intervention. Teng [120] extracted crude *P. Cyrtonema* and steam-processed *P. cyrtonema* from *Polygonatum cyrtonema *in vitro using different methods, and the results showed that both polysaccharides had free radical scavenging activity in vitro. Ha et al. [121] extracted seven steroidal saponins from *Polygonatum*, which were further found to significantly inhibit nitric oxide activity in lipopolysaccharide-activated cells.

#### In vivo study

In animal experiments, Li et al. discovered that the extract of *Polygonatum kingianum* could effectively prolong the survival time of hypoxic SD rats, increase the expression levels of SOD, glutathione peroxidase (GSH-Px), and catalase (CAT), and decrease the expression levels of MDA, LDH, and creatine kinase (CK) in the myocardial tissues of SD rats. The anti-hypoxic effect of *Polygonatum kingianum* may be related to the scavenging of excess free radicals and inhibition of lipid peroxidation [12]. In an in vivo antioxidant assay, PSPs at doses of 200 and 400 mg/kg/d were found to significantly reduce ROS and MDA and increase SOD levels in a mouse model of cardiac senescence induced by D-Gal (500 mg/kg/d), indicating that PSPs are able to protect myocardial tissues by exerting antioxidant capacity [18].

In addition, it was shown that PSPs attenuated myocardial fibrotic injury in autoimmune myocarditis (AM) rats, demonstrating that PSPs decreased myocardial tissue MDA content and serum levels of tumor necrosis factor-α (TNF-α), Interleukin-6 (IL-6), and transforming growth factor beta1 (TGF-β1), and increased myocardial tissue Janus kinase phosphorylation /Janus kinase(p-JAK/JAK), STAT3 phosphorylation/signal transducer and activator of transcription 3 (p-STAT3/STAT3), SOD content and levels, suggesting that PSPs inhibit oxidative stress and inflammatory responses in AM rats through activation of the JAK/STAT3 signaling pathway [122]. In a mouse model of oxidative damage, treatment with PSPs significantly reversed histopathological alterations, decreased ROS accumulation, increased antioxidant enzyme activities, and promoted nuclear translocation of Nrf2 by decreasing Keap-1 expression and increasing HO-1 expression. It is suggested that this antioxidant activity of *Polygonatum* is closely related to the activation of the NRF2/HO-1 pathway [120, 123].

### Lipid and glucose regulation, and anti-atherosclerosis

Hyperglycemia and dyslipidemia are widely recognized as significant contributors to cardiovascular disease [124], specifically in the pathogenesis of atherosclerosis [125]. Research has demonstrated that abnormalities in lipoproteins play a critical role in driving the development of atherosclerotic CVDs [126]. Consequently, the identification of effective drugs and therapeutic targets for early intervention in atherosclerosis holds great importance in reducing mortality. Several studies have highlighted that *Polygonatum* and its active ingredients, such as polysaccharides, flavonoids and saponins, have hypoglycemic and lipid-lowering activities, and have shown better efficacy in the treatment of atherosclerosis [127, 128].

#### In vitro study

PSPs was reported to reduce lipid accumulation and diminish the expression level of inflammatory factors in insulin-resistant cell models by regulating microRNA-140-5p/interleukin-1 receptor-associated kinase 3 (miR-340-3p/IRAK3), which plays a crucial role in inhibiting atherogenesis [129]. Different from the lipid-lowering effect of PSPs, other studies have revealed that steroid saponins and homoisoflavones extracted from *Polygonatum* have potential hypoglycemic effects [130–132], and the latter has been found to be able to inhibit the formation of advanced glycosylation end products [131]. Recent studies demonstrated that homoisoflavones were also effective glucose transporter 2 (GLUT 2) inhibitors [133], providing a novel mechanism for the hypoglycemic properties of *Polygonatum*.

#### In vivo study

Zhu et al. [134] investigated the role of PSPs in atherosclerosis by feeding adult golden hamsters an atherosclerotic diet containing 1.5 mL of olive oil, 8 mg (3,200,000 IU) of vitamin D2, and 40 mg of cholesterol for 60 consecutive days to construct an atherosclerosis model. Subsequent intervention with PSPs for 60 days showed significant amelioration in serum lipid profile, lipoproteins, and endothelial dysfunction parameters, as well as normalization of the morphology of the aorta and myocardial tissues compared with the model group. Ye et al. [25] constructed a ApoE^−/−^ high-fat mouse model and intervened with PSPs. Compared to the control group, PSPs significantly suppressed the expression of serum lipids including Low-Density Lipoprotein Cholesterol (LDL-C), total cholesterol (TC), and triglycerides, as well as cell adhesion molecules including vascular cell adhesion molecule-1 (VCAM-1) and intercellular adhesion molecule-1 (ICAM-1). Histomorphometric analysis revealed that PSPs reduced aortic lipid accumulation and mitigated aortic intimal hyperplasia and inflammatory cell infiltration. These effects may be attributed to the inhibition of the TLR4/MyD88/NF-κB signaling pathway. Yang et al. [135] found similar effects of PSPs in an atherosclerosis model in rabbits. In addition, *Polygonatum* alleviated lipid metabolism disorders in rats by mediating a network of miRNAs pivoting on the miR-484-Bacteroides/Roseburia axis to prevent hyperlipidemia and atherosclerosis [136].

Consistent with the results of in vitro studies, in vivo experiments also confirmed the hypoglycemic properties of saponins in *Polygonatum.* It can significantly alleviate insulin resistance of diabetic mice [137]. It was also reported that the combination of *Polygonatum sibiricum* saponin and probiotics had a better hypoglycemic effect on diabetic mice, bringing more benefits [138]. The reduction of serum glucose could prevent the occurrence of CVDs such as atherosclerosis to a certain extent [139].

### Anti-inflammation and anti-myocardial fibrosis

Inflammation and myocardial fibrosis are recognized as critical factors in the development of cardiovascular disease, ultimately leading to the onset of heart failure [140–143]. On the one hand, during myocardial injury, irrespective of the initiating cause, aseptic myocardial inflammation occurs, which in turn initiates an elevation in the levels of circulating inflammatory factors [144]. On the other hand, the extracellular matrix (ECM) provides a framework for the cardiac myocytes, thereby ensuring the structural and functional integrity of the heart [145]. However, excessive accumulation of ECM in the cardiac interstitium leads to fibrous remodeling of the myocardium, causing contractile dysfunction of the myocardium [146, 147]. In injured myocardial tissues, inflammation and fibrosis often coexist, with substantial infiltration of inflammatory factors observed in the fibrotic myocardium [148]. Studies have demonstrated that *Polygonatum* exerts its effects by attenuating inflammatory response and fibrosis through the modulation of multiple signaling pathways, including the TLR4-MyD88-NF-kappa B and AMPK-Nrf2 pathways [16, 149–151].

#### In vitro study

Zhao et al. [152] identified 27 active components through chemical studies of *Polygonatum sibiricum* Red. Led. Thirteen of these compounds were subsequently investigated for their anti-inflammatory activities, and the results showed that among the 13 compounds, compound β-carboline significantly inhibited the expression of Nitric Oxide (NO), TNF-α, IL-6, and interleukin-1 beta (IL-1β) in lipopolysaccharide (LPS)-treated RAW264.7 macrophages and suppressed the activation of NF-κB, suggesting that compound β-carboline has effective anti-inflammation activities. Lei constructed an H/R model in H9c2 cardiomyocyte in vitro and found that after PSPs intervention, the cell survival rate was significantly increased, the content of inflammatory cytokines and the expression of NF-κB protein were significantly decreased, the expression of inhibitor κB (I-κB) protein was increased, and the level of mRNA expression of TLR4 and MyD88 was significantly decreased, as compared with that of the model group. It is suggested that PSPs may protect H9c2 cardiomyocytes from H/R injury by inhibiting the TLR4-MyD88-NF-κB pathway [153].

#### In vivo study

Yin et al. constructed an autoimmune myocarditis model in SD rats and applied PSPs as an intervention. The results showed that PSPs reduced the levels of TNF-α, IL-6, and TGF-β1, and attenuated myocardial pathological injury and fibrosis in rats. These effects may be attributed to the activation of the JAK/STAT3 signaling pathway [122]. In a study conducted by Zhang et al. [154] using diabetic cardiomyopathic rats, PSPs were administered as an intervention, resulting in a significant improvement in myocardial tissue fibrosis. The mechanism underlying this improvement may be associated with the mediation of the TGF-β1/Smads signaling pathway. Similarly, other researchers have obtained similar conclusions in a rat model of diabetic cardiomyopathy, demonstrating that PSPs can restore the morphology of myocardial tissues and attenuate myocardial fibrosis. These effects may be mediated through the inhibition of the Nod-like receptor protein 3 (NLRP3) inflammasome [155, 156]. In myocardial infarction rats, PSPs were found to improve myocardial injury, reduce inflammatory response, and aid in the repair of ischemic myocardium in acute myocardial infarction rats. The mechanism underlying these effects may be associated with the modulation of NF-κB-mediated inflammatory response [157]. Furthermore, Hirai et al. [158] demonstrated that the methanolic extract of *Polygonatum* exhibited a cardiotonic effect. They observed an increase in the pressure in the left atrium of rats following the administration of the extract. Additionally, the extract effectively inhibited the activity of cyclic adenosine monophosphate (cAMP). The cardiotonic effect was found to be induced by the activation of sympathetic nerves and stimulation of β-adrenergic receptors.

### Amelioration of drug cardiotoxicity

Drug cardiotoxicity refers to the adverse effects of drugs that result in myocardial injury, arrhythmias, abnormalities in cardiac function (systolic or diastolic), cardiac hypertrophy, and in severe cases, heart failure [159]. This issue has become particularly common in the field of oncologic cardiology, where cancer treatments can have detrimental effects on the heart [160–162]. Typical indicators of cardiotoxicity include decreased left ventricular ejection fraction, reduced overall longitudinal strain compared to baseline, and elevated levels of myocardial injury markers such as cardiac troponin T (cTnT), Brain Natriuretic Peptide (BNP), and N-Terminal Pro-Brain Natriuretic Peptide (NT-proBNP) [163]. Cardiotoxicity associated with traditional cancer treatments, such as those caused by chemotherapeutic agents, targeted agents, and the emerging interest in immune checkpoint inhibitor cardiomyopathy, lacks therapeutic agents, and the prevention and treatment strategies have significant room for optimization [162].

Cardiotoxicity is a crucial factor that limits the use of certain drugs [164, 165]. For instance, some chemotherapeutic drugs like anthracyclines carry the risk of dose-dependent cardiotoxicity, increasing the likelihood of heart failure and restricting their clinical application [166]. The mechanism underlying chemotherapy drug-induced cardiotoxicity is highly complex and involves mitochondrial damage, oxidative stress, and cell death. It is a multifactorial interaction of various factors contributing to the development and progression of cardiotoxicity [167–169]. Some previous studies have shown that among some natural products and herbal extracts, tanshinone IIA, salicylic acid, ginsenoside Rg3, ginkgolide B, curcumin, resveratrol, and hexane/ethanol extract of licorice can inhibit ROS production by decreasing mitochondrial lipid peroxidation in cardiomyocytes, inhibiting cardiomyocyte apoptosis, increasing antioxidant activity, and inhibiting inflammation, thus antagonize chemotherapy-induced structural damage in cardiomyocytes [170–172].

*Polygonatum* has been found to exhibit a protective effect against pharmacological cardiotoxicity. Zhu et al. [17] administered PSPs to rats at doses of 100, 200, and 400 mg/kg for 5 days, followed by an intraperitoneal injection of adriamycin on day 6 to induce acute heart failure (AHF). The results showed that compared to the model group, the administration of PSPs significantly improved cardiac function. Additionally, the levels of serum myocardial injury markers cTnT and creatine kinase-MB (CK-MB) were significantly decreased. PSPs also elevated the levels of myocardial Na^+^-K^+^-ATPase, Ca^2+^-Mg^2+^-ATPase, succinate dehydrogenase, and B-cell lymphoma-2 (Bcl-2) protein expression. Moreover, PSPs reduced the levels of TNF-α, IL-6, MDA, and NO, as well as the protein expression of myocardial BCL2-Associated X (Bax) and cleaved caspase-3, indicating a significant reduction in cardiomyocyte apoptosis. These findings suggest that PSPs can prevent adriamycin-induced AHF and the mechanism may be attributed to their anti-oxidative stress, anti-inflammatory, and anti-cardiomyocyte apoptosis properties. Dioscin is the predominant active ingredient in *Polygonatum* saponins. Zhao et al. [88] investigated the efficacy and mechanism of dioscin against drug cardiotoxicity, another active ingredient found in *Polygonatum*. They established in vivo and in vitro models of doxorubicin (DOX)-induced myocardial injury, and the results demonstrated that dioscin increased the viability of H9c2 cells, reduced the expression levels of CK and LDH, and improved histopathological changes and cardiac function in rats and mice exposed to DOX. Furthermore, dioscin exhibited significant inhibition of myocardial oxidative damage both in vitro and in vivo. In addition, dioscin activated the Nrf2 and Sirt2 signaling pathways, leading to the modulation of HO-1, Quinone Oxidoreductase 1 (NQO1), glutathione-S-transferase (Gst), glutamate cysteine ligase modifier subunit (GCLM), Keap1, and Forkhead box O3 (FOXO3a) expression. Moreover, it decreased the expression level of miR-140-5p. These findings suggest that dioscin mitigated DOX-induced cardiotoxicity by regulating miR-140-5p-mediated myocardial oxidative stress. In a word, the active ingredient found in *Polygonatum* shows great potential for application in the field of cardiotoxicity, which can be developed and utilized as a potential candidate for future drug development.

## Discussion

*Polygonatum*, as a traditional herbal medicine, has a long history of medicinal use and precise clinical efficacy and has a broad development prospect. Nowadays, thanks to the rapid development of separation means and structure identification techniques, people have carried out in-depth studies on the active ingredients of *Polygonatum*, optimized and identified its extraction process and chemical composition, and further clarified the pharmacokinetic characteristics of *Polygonatum*’s to promote the application and promotion of the drug. Polysaccharides, flavonoids, and saponins are the three main constituents studied most in *Polygonatum*, while research reports on the other trace constituents are not deep enough. In addition, the biological activities of the various chemical ingredients of *Polygonatum* need to be further developed, which is of great significance in exploring its pharmacological effects.

In this review, we systematically summarized the pharmacological effects and underlying mechanisms of *Polygonatum* and its main active ingredients (Polysaccharides, Flavonoids, Saponins) in the prevention and treatment of CVDs. *Polygonatum* possesses significant antioxidant activity and can play a cardioprotective role by activating NRF2/HO-1 and JAK/STAT3 signaling pathways [120–123]. *Polygonatum* has also been shown to attenuate myocardial fibrosis and inflammation and restore the morphology and function of myocardial tissue by regulating AMPK-Nrf2, NF-κB, TGF-β1/Smads, and NLRP3 inflammasome signaling pathway [152–157]. In addition, *Polygonatum* also has significant efficacy in lipid regulation and anti-atherosclerosis, which can prevent and control the occurrence of atherosclerosis by regulating metabolic abnormality, reducing lipid accumulation, inhibiting hyperglycemia, and reducing vascular damage, so as to intervene in the risk factors of CVDs [129–139]. Atherosclerosis is closely related to hyperlipidemia, inflammation, and oxidative stress, so it is clear that the pharmacological effects exerted by *Polygonatum* are intertwined.

Impressively, *Polygonatum* is demonstrated to have protective effects against cardiotoxicity induced by anticancer therapy and other compounds [17, 88], which is a unique highlight of *Polygonatum* in preventing and treating CVDs. In the future, it is necessary to further elucidate the underlying pharmacological mechanism to promote the application of *Polygonatum* in the field of Cardio-Oncology. In summary, the existing studies suggest that *Polygonatum* may be a potential therapeutic agent for CVDs, and further clarification of the pharmacological mechanisms of *Polygonatum* and its monomer ingredients or active sites are necessary, so as to provide a more rigorous and systematic basis for clinical applications.

Toxicological studies of drugs are an important part of the process of clinical use. Existing studies have shown that *Polygonatum* can reduce its volatile components after concoction, which plays a certain role in reducing toxicity. Some of the acute and chronic toxicity studies done on animals have also shown that *Polygonatum* has very low toxicity and did not cause serious toxicity or death in animals, and no genotoxicity was found either. However, there is still a great lack of high-quality studies on the clinical application, toxicity and side effects of *Polygonatum*, so it is difficult to determine the possible chronic accumulation caused by long-term use of *Polygonatum*. Further studies are still needed to determine the optimal therapeutic dose, safe dosing range, and recommended duration of *Polygonatum*. This suggests that the toxicologic evaluation of *Polygonatum* must be fully considered in future studies in the pharmacologic study of *Polygonatum*. Therefore, further studies are needed to establish an accurate, rapid, reliable, and sensitive modern method of analyzing toxic components to mitigate toxicity to ensure its safe use.

## Conclusions and prospects

In recent years, significant progress has been made in the research on the application of *Polygonatum* and its active ingredients in cardiovascular diseases. Based on the available evidence, the active ingredients of *Polygonatum*, including polysaccharides, flavonoids, and saponins, have shown potential cardioprotective effects through various mechanisms such as anti-oxidative stress, anti-inflammation, anti-fibrosis, lipid regulation, and anti-atherosclerosis. Moreover, *Polygonatum* exhibits a broad therapeutic scope for drug-induced cardiotoxicity. Despite these promising findings, there are several limitations in the current research on *Polygonatum*. Firstly, many pharmacological mechanisms of *Polygonatum* in the prevention and treatment of cardiovascular diseases remain unclear, and the existing studies are relatively isolated, emphasizing the need for more extensive and in-depth research in the future. Secondly, most pharmacological studies have been conducted at the animal and cellular levels, with limited evaluation of its clinical applications and a lack of high-quality evidence-based medical evidence. Therefore, further studies are necessary to validate the potential cardioprotective effects of *Polygonatum* in patients with cardiovascular diseases. *Polygonatum*, as a traditional Chinese medicine with a long history of clinical use, requires more rigorous investigations into its active ingredients, pharmacological mechanisms, pharmacokinetics, and toxicology. These studies are crucial for the wider adoption and modernization of Chinese medicine.

## Data Availability

Not applicable.

## Abbreviations

AHF: Acute heart failure
AM: Autoimmune myocardioptis
AMPK: Adenosine 5ʹ-monophosphate (AMP)-activated protein kinase
ApoE: Apolipoprotein E
ATP: Adenosine triphosphate
Bax: BCL2-Associated X
Bcl-2: B-cell lymphoma-2
BNP: Brain Natriuretic Peptide
cAMP: Cyclic adenosine monophosphate
CAT: Catalase
CK: Creatine kinase
CK-MB: Creatine kinase-MB
cTnT: Cardiac troponin T
CVDs: Cardiovascular diseases
D-gal: D-galactose
DOX: Doxorubicin
DPPH: 2,2-Diphenyl-1-picrylhydrazyl
ECM: Extracellular matrix
FOXO3a: Forkhead box O3
GCLM: Glutamatecysteine ligase modifier subunit
GLUT 2: Glucose transporter
GSH: Glutathione
GSH-Px: Glutathione peroxidase
GSS: Glutathione synthetase
Gst: Glutathione-S-transferase
H/R: Hypoxia/reoxygenation
HO-1: Heme oxygenase-1
HPLC–MS: High Performance Liquid Chromatography-Mass Spectrometry
ICAM-1: Intercellular adhesion molecule-1
IL-1β: Interleukin-1 beta
IL-6: Interleukin-6
IRAK3: Interleukin-1 receptor-associated kinase 3
I-κB: Inhibitor κB
Keap1: Kelch-1ike ECH-associated protein l
LDH: Lactate dehydrogenase
LDL-C: Low-Density Lipoprotein Cholesterol
Lp(a): Lipoprotein(a)
LPS: Lipopolysaccharide
MDA: Malondialdehyde
miR-140-5p: MicroRNA-140-5p
miR-340-3p: MicroRNA-340-3p
MyD88: Myeloid differentiation factor88
NF-κB: Nuclear factor kappa-B
NLRP3: Nod-like receptor protein 3
NO: Nitric Oxide
NQO1: NADPH: Quinone Oxidoreductase 1
Nrf2: Nuclear factor erythroid 2-related factor
NT-proBNP: N-Terminal Pro-Brain Natriuretic Peptide
p-JAK/JAK: Janus kinase
PSP: *P**olygonatum sibiricum* Polysaccharides
p-STAT3/STAT3: STAT3 phosphorylation/Signal Transducer And Activator Of Transcription 3
ROS: Reactive oxygen species
SOD: Superoxide dismutase
TC: Total cholesterol
TGF-β1: Transforming growth factor-beta1
TLR4: Toll-like receptor4
TNF-α: Tumor necrosis factor-α
VCAM-1: Vascular cell adhesion molecule-1
